# Editorial Brevities

**Published:** 1887-11

**Authors:** 


### EDITORIAL BREVITIES.

LIBERAL OFFER.—To each subscriber who, on renewing his subscription, will send us a new subscriber, we will deduct fifty cents on each name; that is for $3.00 we will furnish the RECORD twelve months to you and your friend.

WELLS & RICHARDSON.—Physicians would do well to examine the advertisement of the above firm. The composition of their *Lactated Food* speaks for itself. It is certainly worthy of trial in that troublesome class of cases so trying to the practitioner with which infants are so often attacked.

PARKE, DAVIS & CO. had a most beautiful display at the Piedmont Exposition, presided over by the polite and indefatigable Dr. Stone. The old adage that "the rolling *stone* gathers no moss" does not apply to him, as he gathers much, and dispenses many choice and elegant things. Let him roll.

JOHN B. DANIEL, WHOLESALE AND RETAIL DRUGGIST.—Read John B. Daniel's advertisement. Mr. Daniel is a polite and good business man. He keeps a good stock of drugs at reasonable rates. His store is at a very convenient and central point, being just opposite the entrance to the general passenger depot. Call and see him.

CALLS.—The following medical brethren favored us by calling at our editorial sanctum during the recent Piedmont Exposition, to-wit: Dr. W. S. Kendall, of Cedar Town, Louisiana; Dr. J. M. McMichael, of Marietta, Texas; Dr. A. Barry, of Ringgold, and Dr. W. L. Green, of Stephens, Georgia.

While we much enjoyed the social pleasure of these visits, we were saddened by the information communicated by one of them that his brother, Dr. C. W. McMichael died on the 10th of October, after a brief illness at his home in Marietta, Texas.

GLEDITSCHINE—WILL ITS CLAIMS BE SUBSTANTIATED?—Dr. J. H. Claiborne, in the *New York Medical Record*, Dr. Herman Knapp, in the same journal, and Dr. Edward Jackson, of the *Philadelphia Medical News*, have recently published the results of their experience with the new local anæsthetic, improperly called stenocarpine, but which, being derived from *gleditschia triaganthos*, should be called gleditschine

If the claims of these writers are substantiated by further experience we may expect cocaine to yield its supremacy to this new claimant for anæsthetic honors. The results recorded would indicate that gleditschine possesses greater anæsthetic power than cocaine, and mydriatic effects exceeding those of homatropine.

Those who desire to subject this drug to clinical trial should address Parke, Davis & Co., who we learn are investigating its claims at their laboratory, and offer samples of the fluid extract with a working bulletin descriptive of the drug, to those physicians interested in testing it, and hope soon to be able to supply the alkaloid itself.

RECEIPTED.—1887—B. E. Clark, M. E. Demaret, M. L. Bell, R. A. Shimpock, J. M. McMichael, Mose Yarbrough, A. R. Jones. J. M. Pinkston, to January, 1887. 1888—J. M. Bean, to April; J. W. Julian, to March; R. M. Bones, to March; L. M. Sloan, to March; R. S. Pelham, to March.

